# Intravital Microscopic Research of Microembolization with Degradable Starch Microspheres

**DOI:** 10.1155/2013/242060

**Published:** 2013-11-13

**Authors:** Micaela Ebert, Juergen Ebert, Gerd Berger

**Affiliations:** ^1^Cardiac Center, University of Freiburg, Südring 15, 79189 Bad Krozingen, Germany; ^2^PharmaCept GmbH, Bessemerstraße 82, 12103 Berlin, Germany; ^3^Department of Surgery, Charité-Universitätsmedizin Berlin, Campus Benjamin Franklin, Hindenburgdamm 30, 12200 Berlin, Germany

## Abstract

Treatment efficacy in cancer patients using systemically applied cytostatic drugs is decreased by cytotoxic side effects, which limits the use of efficient dosages. Degradable starch microspheres (DSM) are used to apply drugs into blood vessels which supply the target organ leading to drug accumulation in the target organ by reduction of the blood flow. The present investigations show that DSM is a very effective embolization material leading to effective and enhanced accumulation of 5-FU within the liver tumor tissue of experimental induced liver cancer in rats. By using intravital microscopy, a rapid deceleration of the blood flow into the target organ is observed immediately after application of DSM. The microspheres are stepwise degraded in the direction of the systemic blood flow and are totally dissolved after 25 minutes. These stepwise processes leave the degraded material during the degradation process within the vessels leading to temporally reciprocal blood flow via some of the side-arms of the major blood vessels. By using DMS in transarterial chemoembolization (TACE), severe adverse side effects like postembolization syndrome are rarely observed when compared to other embolization materials. The complete degradation of DSM causes only a short-lasting temporary vascular occlusion, which allows a repeat application of DSM in TACE.

## 1. Introduction

Systemic chemotherapy in cancer patients with liver tumors or liver metastases shows up to now especially with respect to the prolongation of overall survival insufficient results probably due to not high enough local tumor drug dosages [1]. Collins and coworkers could show that the response rates can be doubled when the drug concentration is increased by a factor of 10 [2]. However, systemic applied cytostatic drugs may worsen the quality of life of patients by sometimes very severe adverse side effects especially when used in high dosages. Those cytotoxic side effects limit the use of efficient dosages. Thus, since several years various techniques were investigated and used for intra-arterial administration of certain cytostatic drugs, which allows higher drug concentrations [3]. It could be shown, for example, that regional infusion of 5-fluorouracil (5-FU) increases liver exposure to the drug by a factor of 100 when compared to intravenous application route [4]. In fact, meanwhile, several randomized clinical trials in colon cancer patients suffering from liver metastases have shown that the intra-arterial application of 5-FU or floxuridine leads to increased response rates with a tendency to prolongation of the overall survival [5–10]. However, the liver is a high blood flow organ receiving a large fraction of the cardiac output leading immediately to transportation of the drug outside the target organ [11]. In this context, reduction of the regional blood flow by occluding the vascular bed when administering the drug is one of the most important factors for an effective drug delivery into the liver tumor via intra-arterial application [12]. Several embolization materials were tested and used to reduce the regional blood flow [3, 11, 12]. However, some of these materials lead to permanent vascular occlusion and thus limit repeated treatments [3, 11, 12]. Meanwhile, the implantation of degradable starch microspheres (DSM) to TACE is accepted by several publications showing the near-term reproducibility, higher accumulation rates of the coapplied drugs, less toxicity though significantly reduced cytotoxic peak plasma concentrations, less postembolization syndrome, and the unique possibilities of combination with drugs and other treatment techniques [3, 11, 13–19]. DSM are produced from partly hydrolysed starch, cross-linked, and substituted with glycerol ether groups and are degradable by *α*-amylase [20]. The complete degradation of DSM by *α*-amylase causes only a short-lasting temporary vascular occlusion, which allows a repeated application of DSM in TACE [3]. Such repeated treatments are highly important to achieve an optimal tumor control. Various cytostatic drugs have been used in combination with DSM, for example, mitomycin C, doxorubicin, epirubicin, or cisplatin, showing a significant better tumor response [12, 13]. Objective clinical response rates up to 88% have been shown by some published clinical trials [21, 22]. More recently Vogl and coworkers published data from 462 patients with liver metastases from colon cancer, which were treated with DSM combined with a cytostatic drug showing an improved median overall survival (38 months) after primary diagnosis compared to reported data using intravenous applied chemotherapy [23]. 

Although several clinical and experimental data are published about the effect of DSM in cancer treatment, it is still not fully clarified what happens within the blood vessel during the degradation processes. The aim of the present study was to visualize and measure the accumulation of 5-FU combined with DSM into healthy liver as well as into liver tumor tissue of experimental induced liver cancer in rats using intravital microscopy and biochemical measurement, respectively. Furthermore, the processes of degradation of DSM are visually monitored in order to get more insight into the mechanism of the degradation to fully understand the resulting increased drug uptake into the liver tumor lesion.

## 2. Material and Methods

To investigate the processes of DSM degradation, DSM based occlusion of blood vessels, and DSM based drug accumulation into healthy liver as well as into experimental liver tumors of rats, the technique of intravital microscopy was used. For optimal visualization, DSM and 5-FU were labelled with fluorescein dye (FITC). 

### 2.1. Experimental Animals

The experimental animals were WAG/Rij-rats (Wistar Albino Glaxo), weighting between 250 and 300 g, at our disposal. They were fed with standard rat food with free access to water. Animals were used in accordance with the national guidelines for the care and use of laboratory animals. 

### 2.2. Experimental Liver Tumor

Experimental rat liver tumors were prepared by using the tumor cell line CC531, which is a moderately differentiated adenocarcinoma originating from the colon of rats exposed to methylazoxymethanol. The ready for injection CC531-adenocarcinoma cells were kindly provided by the study group “drug targeting,” Max-Delbrück-Centre for molecular-diagnostic medicine, Berlin-Buch [24]. The tumor cells were implanted into the rat liver, which is an established model for human liver metastasis of colorectal carcinoma. 12 hours prior to tumor cell implantation rats were restrained from food. Anaesthesia was performed by intramuscular injection of Xylazine (12 mg/kg) and Ketamine-HCL (80 mg/kg). Additional anaesthesia was given intravenously, whenever necessary. Subsequently, the rats were dissected by a midline abdominal incision through the linea alba. After having retracted the left liver lobe, the tumor cell suspension (7.5 × 10^5^ cells per rat) was injected through a cannula (0.45 × 15 mm), strictly subcapsular. The penetration mark was sealed with tissue glue (Histoacryl, B. Braun Surgical GmbH, Melsungen, Germany). Infection prophylaxiswas performed by intraperitoneal administration of 0.03 mL Penicillin-G (Penicillin “Grünenthal”, 1 Mega; Grünenthal GmbH, Stolberg, Germany) and Streptomycin (Streptomycin-Heyl 1 g; Heyl Chem.-pharm. Fabrik GmbH, Berlin, 3 Germany). After approximately 12–16 days, the animals developed tumors with 1–1.5 cm extent.

### 2.3. Application of DSM and 5-FU

DSM (Spherex, Pharmacia, Erlangen, Germany) alone or in combination with the chemotherapeutic drug 5-FU were injected into the blood vessel which supplies the rat liver. To evaluate the normal blood flow, erythrocytes were marked by a fluorescent dye by injection of 12 mg in 0.2 mL of 1 g/L fluorescent sodium 0.1 mL into the cannulised gastroduodenal artery (GDA), the proper hepatic artery. DSM as well as 5-FU were also FITC-labelled and were likewise injected. 

### 2.4. Intravital Microscopy

The gastroduodenal artery (GDA) as well as the smaller peripheral vessels was exposed to the liver surface by retraction of the left liver lobe. During the procedure, the surface of the liver lobe was covered with a small piece of plastic wrap and constantly rinsed with ringer solution at body temperature. The retracted left liver lobe was carefully placed under a fluorescent microscope and transilluminated with monochromatic light, generated by a prism monochromator equipped with xenon light. The in vivo microscopic investigations were monitored and recorded on videotapes. 

### 2.5. Measurement of 5-FU Concentration

The accumulation rates of 5-FU within the liver and liver tumor applied with or without Amilomer, DSM, were biochemically measured by HPLC. Before measurement, the liver with and without tumor was homogenized. The HPLC analyses were performed as described by Pohlen and coworkers at room temperature [24]. Therefore, 5 animals each were killed 15, 30, 60, 90, 120, and 240 minutes after i.a therapy with 5-FU and without DSM being started, and the 5-FU concentrations in different organs were determined by HPLC. An additional time point (480 min) was selected in the therapy group 5-FU with DSM. Results were graphically visualized by area under the concentration time curve (AUC). 

## 3. Results

### 3.1. Blood Flow and DSM Induced Occlusion

**Figure 1:**
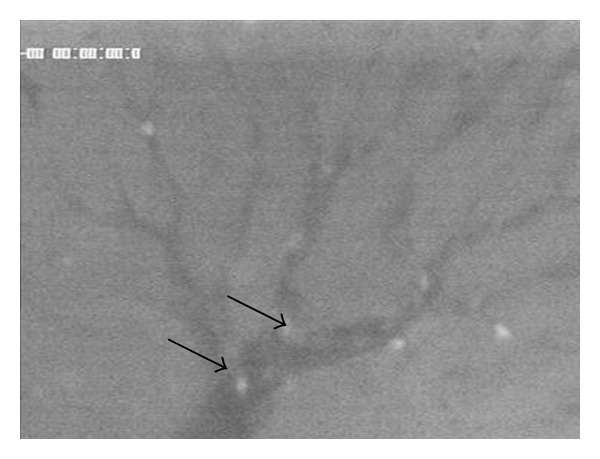
Microcirculation of the rat liver blood flow by visualization of the FITC-labelled erythrocytes.

**Figure 2:**
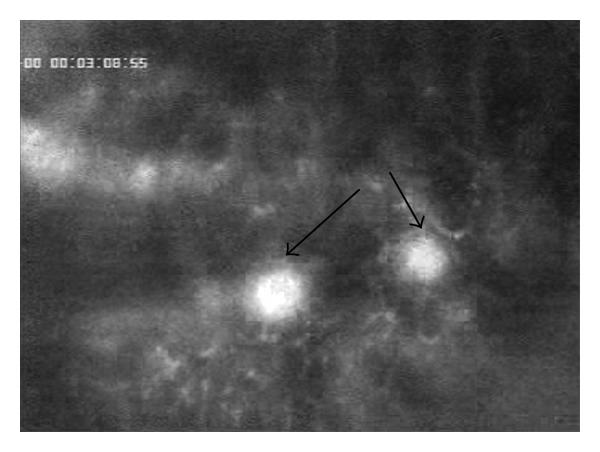
Occlusion effect of FITC-labelled DSM.

Figure 1 shows the normal microcirculation of the liver blood flow by visualization of the FITC-labelled erythrocytes. Injection of FITC-labelled DSM leads to occlusion of the microcirculation (Figure 2). DSM is mainly found in the central sites of the target organ. Furthermore, it can be found in peripheral tumor areas leading to occlusion of the microcirculation around the tumor margin. Thus, the blood flow can be stopped temporarily in the whole organ. 

Infusion of 5-FU by intra-arterial application combined with DMS shows an increased drug accumulation within the tumor tissue compared to the normal liver parenchyma. This can be also demonstrated by biochemical measurement showing that the AUC in the targeted tumor tissue is 95 times higher when 5-FU is applied in combination with DMS.

### 3.2. Degradation of DSM and the Resulting Effects on the Blood Flow

**Figure 3:**
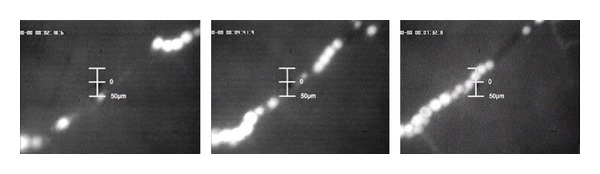
Stepwise occlusion of the blood vessel by accumulation of FITC-labelled DSM.

As shown in Figure 3, DSM accumulates within the arterioles and blood vessels immediately after DMS is injected into the hepatic artery leading to stepwise occlusion of the vessel. After approximately 8 minutes, the blood vessel is completely occluded.

**Figure 4:**
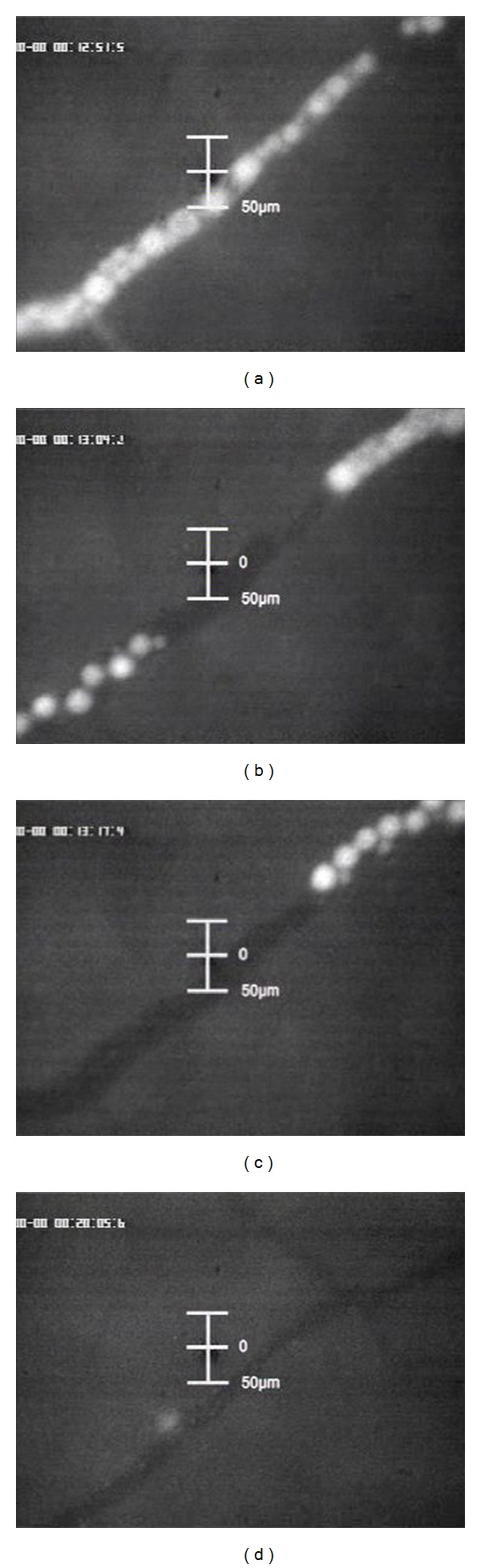
(a) Diffuse contours of particles. (b) Partly washout of particles. (c) Washout of remaining particles along with the systemic blood circulation. (d) Reestablishing of the normal physiological blood flow.

First sign of DSM degradation process can be observed after approximately 7–13 minutes (Figure 4(a)). The contours of the particles become more diffuse and the FITC-labelled degraded material is eliminated by washout (Figure 4(b)). Shortly afterwards, the remaining still intact but smaller particles are washed out along with the physiological blood flow in direction of the capillary bed and the systemic blood circulation (Figure 4(c)). After round about 25–40 minutes, all starch microspheres are dissolved and no DSM particles are visible. The physiological blood flow has completely turned to normal (Figure 4(d)).

**Figure 5:**
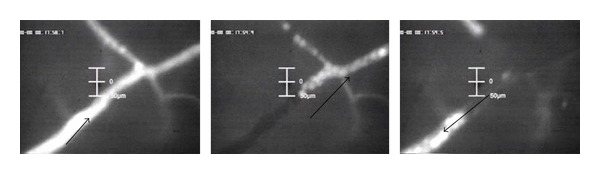
Forward and backward movements of the blood flow while the degradation process of DSM is proceeding.

Interestingly, the degradation processes of DSM lead to temporally blood flow shiftings caused by a negative pressure in the occluded blood vessels (Figure 5). The blood flow movements are supposed to be mainly caused by the degradation mode of *α*-amylase leading to randomly and stepwise degradation of the microspheres. Furthermore, the particles are designed to maintain their spherical shape until they are completely dissolved [25]. These stepwise processes leave the degraded material during the degradation process within the blood vessels. Due to the increasing arterial pressure and due to the persisting occlusion effect of DSM, the blood flow centralizes in diverse side-arms of the precapillary system. Thereby, a negative pressure is created and may lead to the temporally reciprocal blood flow via some of the side-arms of the major blood vessels. These forward and backward movements happened several times even in peripheral tumor areas leading to increased contact frequency of the drug with the tumor tissue.

### 3.3. Intra-Arterial Chemotherapy with 5-FU with and without DSM Occlusion

**Figure 6:**
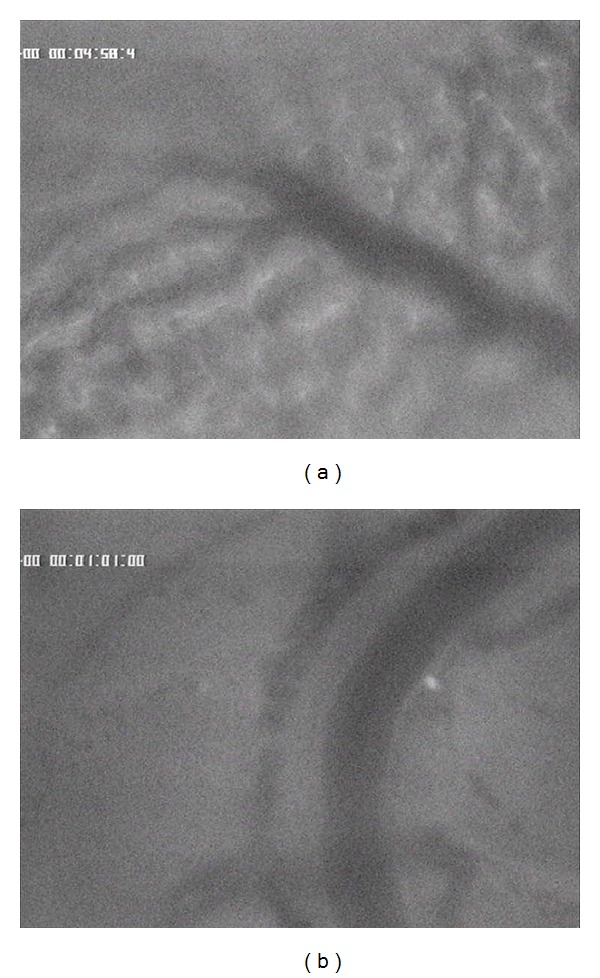
(a) 5-FU accumulation in healthy liver (blood vessels are dark). (b) 5-FU accumulation in liver tumor.

After application of FITC-labelled 5-FU without combination of DSM, the chemotherapeutic agents diffuse immediately and accumulate within a few minutes into the healthy liver parenchyma (Figure 6(a)). The blood vessels appear dark. By using the same dosage of the cytostatic drug, FITC-labelled 5-FU accumulates in a slightly higher concentration into liver tumor parenchyma (Figure 6(b)). But in contrast to normal parenchyma, the normal liver reticulation cannot be visualized due to the equable diffusion of the drug (Figure 6(b)).

**Figure 7:**
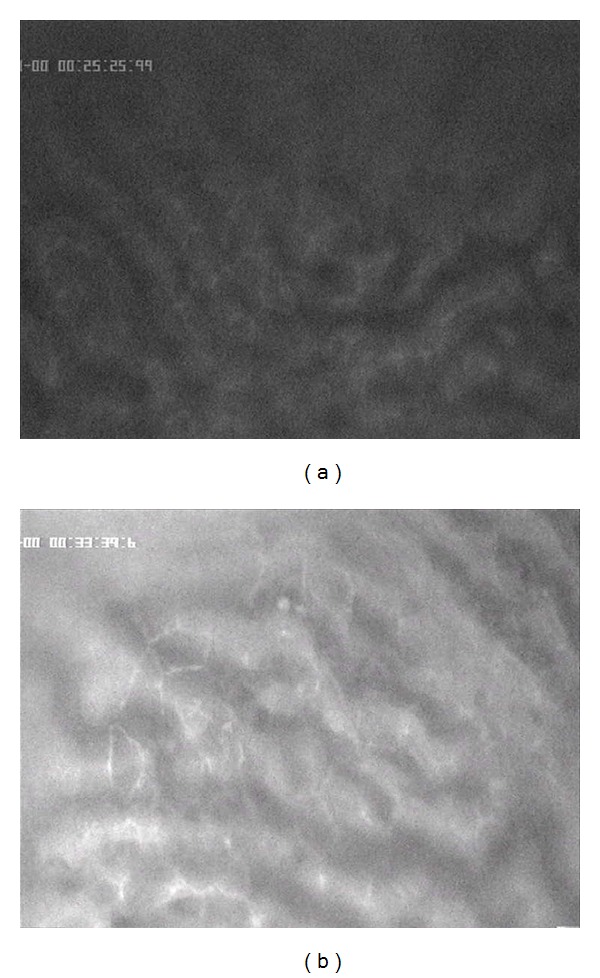
5-FU accumulation in healthy liver parenchyma without (a) and with chemo-occlusion through DSM (b) after 25 minutes.

After approximately 25 min, the 5-FU concentration is still constant visible in the healthy liver parenchyma with or without DSM (Figure 7). The pharmacologically proven small differences between the concentration rates of an applied drug combined with or without DSM [15] are not clearly visible due to the very small differences in the concentration rates.

**Figure 8:**
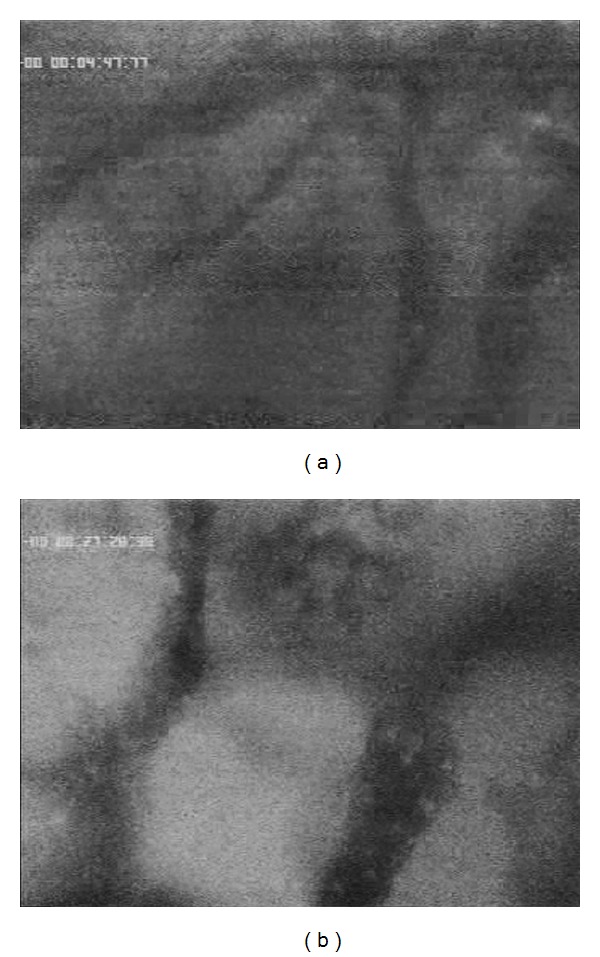
5-FU accumulation in liver tumor without (a) and with chemo-occlusion through DMS (b) after 25 minutes.

However, in liver tumor tissue, the differences in the 5-FU accumulation rates in relation to combination of DSM are, even after 25 minutes, clearly visible (Figure 8). The 5-FU accumulates in higher intensity with coapplication of DSM (Figure 8(b)) than without chemo-occlusion (Figure 8(a)). 

### 3.4. 5-FU Concentration in Healthy Liver and Liver Tumor with and without DSM

**Figure 9:**
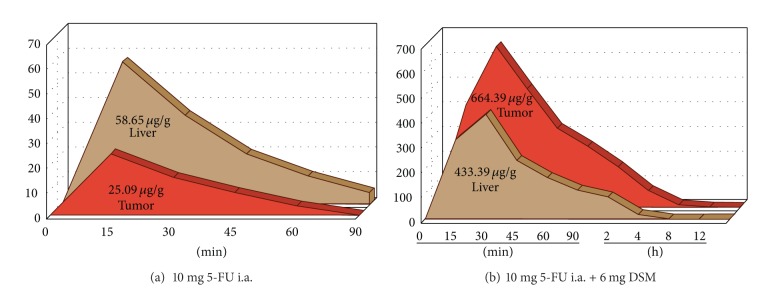
5-FU accumulation (AUC curve) in healthy liver parenchyma and liver tumor tissue without (a) and with chemo-occlusion through DSM (b).

In healthy liver parenchyma as well as in liver tumor tissue, the accumulation rates of 5-FU are increased when DSM is combined (Figure 9). Furthermore, the pharmacokinetics of 5-FU were changed. The peak level after intra-arterial infusion of 5-FU alone was in the healthy liver parenchyma 58.65 *μ*g/g and in the tumor tissue 25.09 *μ*g/g. The concentration maximum was reached after approximately 15 minutes (Figure 9(a)). When combined with DSM, the peak level of 5-FU was 433.39 *μ*g/g in the healthy liver parenchyma and 664.39 *μ*g/g in the tumor tissue. The concentration maximum of 5-FU was reached approximately 30 minutes after intra-arterial infusion with DSM (Figure 9(b)). 5-FU in liver tissue was still measurable 12 hours after administration when combined with DSM compared to only 90 minutes when applied without DSM (Figure 9). 

The therapy group 5-FU with DSM demonstrated significantly higher 5-FU concentrations (*P* < 0.01) compared to the intra-arterial group 5-FU alone. In group 5-FU alone i.a. the 5-FU AUC in the healthy liver parenchyma and tumor tissue measured at the time points from 15 to 240 min was 1704 *μ*g/g and 655 *μ*g/g, respectively. The highest concentrations were measured after the administration of 5-FU combined with DSM (AUC 15–480 min) 62655 *μ*g/g in tumor tissue compared to (AUC 15–480 min) 27822 *μ*g/g in the healthy liver tissue.

**Figure 10:**
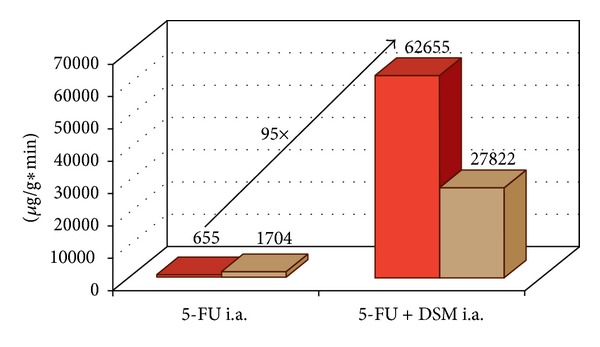
5-FU accumulation (AUC 15–240 min) in healthy liver and liver tumor without and with chemo-occlusion through DSM.

The intra-arterial infusion of 5-FU along with DSM led to a 95 times higher AUC in the targeted tumor tissue. The even observed increased AUC in the healthy liver was clearly less intense (Figure 10). 

## 4. Discussion

Intra-arterial administrations of a cytostatic drug are used to expose the tumor to higher drug concentration without having an increased toxicity to the patients. Several publications have shown in clinical [12, 13, 16, 21–23, 26–28] as well as in pharmacological studies [2, 5, 11, 17] or see above our own unpublished data that DSM within TACE is an effective treatment especially in palliative settings of patients with primary liver cancer or hepatic metastases. The use of DSM in TACE has been shown to improve the time to progress as well as the overall survival of treated patients with primary liver cancer in a phase III clinical trial published by Taguchi and coworkers in 1992 [26]. Similar results were published by Vogl and coworkers in 2009 [23] and Pohlen and coworkers in 2006 [27] for patients with liver metastasis of colorectal cancer. 

The use of DSM to TACE is meanwhile accepted to lead to higher accumulation rates of the coapplied drugs and less toxicity through significantly reduced cytotoxic peak plasma concentrations. For example, Andersson et al. [15] could show that combining DSM with mitomycin C reduces the systemic exposure of the chemotherapeutic drug leading to less hematologic toxicity. Furthermore they showed that the area under the concentration time curve (AUC) in treated patients was significantly lower when the drug was coadministrated with DSM, while the terminal half-life (*t*_1/2_) of mitomycin C was unchanged [15]. Beside mitomycin several other chemotherapeutic drugs like 5-FU [27], gemcitabine [28], or doxorubicin [26] can be used along with DSM within TACE in order to significantly enhance the accumulation of the drug into the target tissue. Moreover, Pohlen and coworkers [24, 29] could show that a liposomal carrier (stealth liposome) used for drug targeting approaches achieved better results in combination with DSM leading to a 2203 times increase of the intratumoral concentration of 5-FU [24, 29]. These previous results are concordant with the results of the present investigation showing the effective and enhanced accumulation of 5-FU within liver tumor tissue when combined with DSM. This could be shown by intravital microscopy as well as by pharmacological analyses. 

Nowadays, a lot of facts are known about the unique way of decelerating the blood flow in DSM filled vessels [30]. Nevertheless, it is yet not fully understood and clarified why especially DSM had beneficial impacts on tumor treatment along with chemoembolization procedures. The present study verified that one of the main reasons for this purpose is the common known embolization material effect—the closing of the main tumor supporting vessels leading to reduction of the regional blood flow. However, several other tested embolization materials can also reduce the regional blood flow [3, 11, 12]. The advantage of DMS compared to other occluding embolization materials may be probably due to its dynamic effects caused by the mechanism of the degradation processes. Here, we could show that the stepwise degradation processes of DSM via *α*-amylase lead to temporally blood-flow-shiftings caused by a negative pressure in the occluded blood vessels. The remaining degraded DSM material as well as the persisting occlusion effect of DSM leads to increasing arterial pressure. As a result, the blood flow centralizes in the side-arms of the precapillary system. As a result a negative pressure is created, which leads to a temporally reciprocal blood flow via some of the side-arms of the major blood vessels. These forward and backward movements happened several times leading to increased contact frequency of the drug within the tumor tissue and thus can explain the advantageous effects of using DSM in TACE. The variability of the arterial blood flow caused by dynamic changes in the DSM degradation processes could also be demonstrated by Civalleri and coworkers [31]. They could show that the use of DSM causes flow redistribution towards the hypovascular areas. When using the drug alone, only very low drug concentrations reach the hypovascular regions in spite of a comparably high initial dose leading to the suggestion that cancer cells within this area may probably lead to disease progression [31]. 

Beside the above described effects, it is well known that the use of DSM causes much less postembolization syndrome than using other common embolization material [32, 33]. By analysing the microscopic pictures of the present study, one can suppose that this effect can also be explained by DSM caused visible dynamic changes within the blood vessels leading probably to a shorter ischemia time laps for the healthy or tumor unaffected tissue. 

The use of DMS in TACE may also give the advantage to combine immune therapeutic treatment approaches. Altomonte and coworkers [34] could, for example, demonstrate that injection of recombinant vesicular stomatitis virus vaccine along with DSM (EmboCept) into the hepatic artery of rats with experimental induced HCC leads to a higher accumulation of the virus into the target organ as well as to a higher level of tumor necrosis and improvement of the survival. Furthermore, a combination of DSM with an adenovirus administered through the hepatic area has been shown to result in an efficient and cancer selective gene transfer [35]. 

During the last decade, immune therapeutic treatment approaches focused mainly on the use of autologous dendritic cells in vaccination strategies in order to induce an antitumor response by activation and induction of tumor-specific cytostatic T cells [36]. However, a lot of patients have a functionally impaired immune system due to the previous applied cytostatic drugs. Several systemic applied cytostatic drugs may lead to immune suppression and reduction of certain immune cells limiting the effect of immune therapeutic treatment strategies. On the other hand, it has been shown that chemotherapeutics can exhibit several beneficial effects on the immune system in spite of its myelosuppressive effects. For example, gemcitabine increases the antigen cross-presentation, T lymphocyte expansion, and the T-cell infiltration of tumors [37] and 5-FU has been described to upregulate tumor antigen expression on colorectal cancer cells [38]. In fact, some pilot clinical trials in cancer patients indicate that the efficacy of anticancer vaccines may be enhanced by chemotherapy [39]. 

Beside our results and published data from other groups showing the advantage of DSM in TACE, a high-class randomized clinical study with respect to a prolongation of the overall survival is still lacking. Those randomized prospective clinical trials choosing the overall survival as primary endpoint will give the chance to discover whether there is a statistically significant survival benefit as well as an improvement of the quality of life for cancer patients receiving regional drug therapy with DSM. Moreover, the stimulatory effect of chemotherapy on tumor immunogenicity without impairing the immune effector cell function may provide a strong rationale to combine TACE using DSM with dendritic cell vaccination procedures. 

## 5. Conclusion

Taken together, the investigations show that DSM is a very effective embolization material leading to effective and enhanced accumulation of 5-FU especially within the liver tumor tissue. The selective increased accumulation of the drug in the tumor tissue is supposed to be partly due to portal washout, which occurs only in healthy liver parenchyma. Interestingly, the degradation processes of DSM lead to temporally blood flow shiftings caused by a negative pressure in the occluded blood vessels. This mechanism may also lead to increased contact frequency of the drug with the tumor tissue. By using DMS in transarterial chemoembolization (TACE), severe adverse side effects like post-embolization syndrome are rarely observed when compared to other embolization materials like Lipiodol or permanent embolization materials. Several of these materials lead to a permanent vascular occlusion and thus limit repeated treatments. The complete degradation of DSM causes only a short-lasting temporary vascular occlusion, which allows a repeat application of DSM in TACE. Meanwhile, it is known that some chemotherapeutics can exhibit several beneficial effects on the immune system in spite of its myelosuppressive effects. For example, 5-FU has been described to upregulate tumor antigen expression on cancer cells. Thus, the use of DSM in TACE can be probably combined with immune therapeutic treatment approaches not having the same myelosuppressive effect as when the drug is administered systemically.

## References

[1] Gieseler F (2000). The dilemma of gastro-enterological oncology: we know a lot but we still achieve too little. *International Journal of Colorectal Disease*.

[2] Collins JM (1984). Pharmacologic rationale for regional drug delivery. *Journal of Clinical Oncology*.

[3] Håkansson L, Hakansson A, Morales O, Thorelius L, Warfving T (1997). Spherex (degradable starch microspheres) chemo-occlusion-enhancement of tumor drug concentration and therapeutic efficacy: an overview. *Seminars in Oncology*.

[4] Ensminger WD, Gyves JW (1984). Regional cancer chemotherapy. *Cancer Treatment Reports*.

[5] Chang AE, Schneider PD, Sugarbaker PH, Simpson C, Culnane M, Steinberg SM (1987). A prospective randomized trial of regional versus systemic continuous 5-fluorodeoxyuridine chemotherapy in the treatment of colorectal liver metastases. *Annals of Surgery*.

[6] Hohn DC, Stagg RJ, Friedman MA (1989). A randomized trial of continuous intravenous versus hepatic intraarterial floxuridine in patients with colorectal cancer metastatic to the liver: the Northern California Oncology Group trial. *Journal of Clinical Oncology*.

[7] Kemeny N, Daly J, Reichman B, Geller N, Botet J, Oderman P (1987). Intrahepatic or systemic infusion of fluorodeoxyuridine in patients with liver metastases from colorectal carcinoma. A randomized trial. *Annals of Internal Medicine*.

[8] Martin JK, O’Connell MJ, Wieand HS (1990). Intra-arterial floxuridine vs systemic fluorouracil for hepatic metastases from colorectal cancer. A randomized trial. *Archives of Surgery*.

[9] Rougier P, Laplanche A, Huguier M (1992). Hepatic arterial infusion of floxuridine in patients with liver metastases from colorectal carcinoma: long-term results of a prospective randomized trial. *Journal of Clinical Oncology*.

[10] Allen-Mersh TG, Earlam S, Fordy C, Abrams K, Houghton J (1994). Quality of life and survival with continuous hepatic-artery floxuridine infusion for colorectal liver metastases. *The Lancet*.

[11] Johansson C (1996). Pharmacokinetic rationale for chemotherapeutic drugs combined with intra-arterial degradable starch microspheres (Spherex). *Clinical Pharmacokinetics*.

[12] Kemeny N, Carr B, Civalleri D (1995). *An Update on Regional Treatment of Liver Tumors. The Role of Vascular Occlusion*.

[13] Taguchi T (1994). Chemo-Occlusion for the treatment of liver cancer. A new technique using degradable starch microspheres. *Clinical Pharmacokinetics*.

[14] Pohlen U, Berger G, Binnenhei M, Reszka R, Buhr HJ (2000). Increased carboplatin concentration in liver tumors through temporary flow retardation with starch microspheres (Spherex) and gelatin powder (Gelfoam): an experimental study in liver tumor-bearing rabbits. *Journal of Surgical Research*.

[15] Andersson M, Aronsen KF, Balch C (1989). Pharmacokinetics of intra-arterial mitomycin C with or without degradable starch microspheres (DSM) in the treatment of non-resectable liver cancer. *Acta Oncologica*.

[16] Kirchhoff TD, Bleck JS, Dettmer A (2007). Transarterial chemoembolization using degradable starch microspheres and iodized oil in the treatment of advanced hepatocellular carcinoma: evaluation of tumor response, toxicity, and survival. *Hepatobiliary and Pancreatic Diseases International*.

[17] Lindell B, Aronsen KF, Nosslin B, Rothman U (1978). Studies in pharmacokinetics and tolerance of substances temporarily retained in the liver by microsphere embolization. *Annals of Surgery*.

[18] Itani K (1990). Effects of degradable starch microspheres in intra-arterial chemotherpay of liver malignancies. *Journal of Kyoto Prefectural University of Medicine*.

[19] Wacker FK, Reither K, Ritz JP, Roggan A, Germer CT, Wolf KJ (2001). MR-guided interstitial laser-induced thermotherapy of hepatic metastasis combined with arterial blood flow reduction: technique and first clinical results in an open MR system. *Journal of Magnetic Resonance Imaging*.

[20] Lindberg B, Lote K, Teder H, Davis SS, Illum L, McVie JG, Tomlinson E (1984). Biodegradable starch microspheres-a new medical tool. *Microspheres and Drug Therapy*.

[21] Schmoll E, Chavan A, Prokop M A new regional immune-chemo-occlusion regimen in liver metastases of colorectal carcinoma.

[22] Civalleri D, Pector J-C, Hakansson L, Arnaud J-P, Duez N, Buyse M (1994). Treatment of patients with irresectable liver metastases from colorectal cancer by chemo-occlusion with degradable starch microspheres. *British Journal of Surgery*.

[23] Vogl TJ, Gruber T, Balzer JO, Eichler K, Hammerstingl R, Zangos S (2009). Repeated transarterial chemoembolization in the treatment of liver metastases of colorectal cancer: prospective study. *Radiology*.

[24] Pohlen U, Reszka R, Buhr HJ, Berger G (2011). Hepatic arterial infusion in the treatment of liver metastases with PEG liposomes in combination with degradable starch microspheres (DSM) increases tumor 5-FU concentration. An animal study in CC-531 liver tumor-bearing rats. *Anticancer Research*.

[25] Mir S, Shuman LS, Wright KC (1984). Alteration in starch microsphere degradation following contrast angiography in the dog kidney. *Investigative Radiology*.

[26] Taguchi T, Ogawa N, Bunke B, Nilsson B (1992). The use of degradable starch microspheres (Spherex) with intra-arterial chemotherapy for the treatment of primary and secondary liver tumours-results of a phase III clinical trial. *Regional Cancer Treatment*.

[27] Pohlen U, Rieger H, Mansmann U, Berger G, Buhr HJ (2006). Hepatic arterial infusion (HAI). Comparison of 5-fluorouracil, folinic acid, interferon alpha-2b and degradable starch microspheres versus 5-fluorouracil and folinic acid in patients with non-resectable colorectal liver metastases. *Anticancer Research B*.

[28] Vogl TJ, Schwarz W, Eichler K (2006). Hepatic intraarterial chemotherapy with gemcitabine in patients with unresectable cholangiocarcinomas and liver metastases of pancreatic cancer: a clinical study on maximum tolerable dose and treatment efficacy. *Journal of Cancer Research and Clinical Oncology*.

[29] Pohlen U, Reszky R, Schneider P, Buhr HJ, Berger G (2004). Stealth liposomal 5-fluorouracil with or without degradable starch microspheres for hepatic arterial infusion in the treatment of liver metastases. An animal study in VX-2 liver tumor-bearing rabbits. *Anticancer Research A*.

[30] Wang J, Murata S, Kumazaki T (2006). Liver microcirculation after hepatic artery embolization with degradable starch microspheres in vivo. *World Journal of Gastroenterology*.

[31] Civalleri D, Scopinaro G, Balletto N (1989). Changes in vascularity of liver tumours after hepatic arterial embolization with degradable starch microspheres. *British Journal of Surgery*.

[32] Wasser K, Giebel F, Fischbach R, Tesch H, Landwehr P (2005). Transarterielle Chemoembolisation von Lebermetastasen kolorektaler Karzinome mit abbaubaren Stärkepartikeln (Spherex). *Eigene Beobachtungen und Literaturübersicht, Radiologe*.

[33] Vogl TJ, Zangos S, Eichler K, Yakoub D, Nabil M (2007). Colorectal liver metastases: regional chemotherapy via transarterial chemoembolization (TACE) and hepatic chemoperfusion: an update. *European Radiology*.

[34] Altomonte J, Braren R, Schulz S (2008). Synergistic antitumor effects of transarterial viroembolization for multifocal hepatocellular carcinoma in rats. *Hepatology*.

[35] Shiba H, Okamoto T, Futagawa Y (2006). Adenovirus vector-mediated gene transfer using degradable starch microspheres for hepatocellular carcinoma in rats. *Journal of Surgical Research*.

[36] Dauer M, Schnurr M, Eigler A (2008). Dendritic cell-based cancer vaccination: quo vadis?. *Expert Review of Vaccines*.

[37] Nowak AK, Robinson BWS, Lake RA (2002). Gemcitabine exerts a selective effect on the humoral immune response: implications for combination chemo-immunotherapy. *Cancer Research*.

[38] Correale P, Aquino A, Giuliani A (2003). Treatment of colon and breast carcinoma cells with 5-fluorouracil enhances expression of carcinoembryonic antigen and susceptibility to HLA-A(∗)02.01 restricted, CEA-peptide-specific cytotoxic T cells in vitro. *International Journal of Cancer*.

[39] Kaufman HL, Lenz H, Marshall J (2008). Combination chemotherapy and ALVAC-CEA/B7.1 vaccine in patients with metastatic colorectal cancer. *Clinical Cancer Research*.

